# Sociodemographic and health-(care-)related characteristics of online health information seekers: a cross-sectional German study

**DOI:** 10.1186/s12889-015-1423-0

**Published:** 2015-01-29

**Authors:** Laura Nölke, Monika Mensing, Alexander Krämer, Claudia Hornberg

**Affiliations:** Department of Environment & Health, Bielefeld University: School of Public Health, Universitätsstraße 25, 33615 Bielefeld, Germany; NRW Centre for Health, Westerfeldstraße 35/37, 33611 Bielefeld, Germany; Department of Public Health Medicine, Bielefeld University, School of Public Health, Universitätsstraße 25, 33615 Bielefeld, Germany

**Keywords:** Information seeking behavior, Internet, Health literacy, SES

## Abstract

**Background:**

Although the increasing dissemination and use of health-related information on the Internet has the potential to empower citizens and patients, several studies have detected disparities in the use of online health information. This is due to several factors. So far, only a few studies have examined the impact of socio-economic status (SES) on health information seeking on the Internet. This study was designed to identify sociodemographic and health-(care-)related differences between users and non-users of health information gleaned from the Internet with the aim of detecting hard-to-reach target groups.

**Methods:**

This study analyzed data from the NRW Health Survey LZG.NRW 2011 (n = 2,000; conducted in North Rhine–Westphalia, Germany, via telephone interviews). Logistic regression analysis was used to examine the determinants of online health information seeking behavior.

**Results:**

68% of Internet users refer to the Internet for health-related purposes. Of the independent variables tested, SES proved to exert the strongest influence on searching the Internet for health information. The final multivariate regression model shows that people from the middle (OR: 2.2, 95% CI: 1.6–3.2) and upper (OR: 4.0, 95% CI: 2.7–6.2) social classes are more likely to seek health information on the Internet than those from the lower class. Also, women are more likely to look for health information on the Internet than men (OR: 1.5, 95% CI: 1.1–2.1). Individuals with a migration background are less likely to conduct health searches on the Internet (OR: 0.6, 95% CI: 0.4–0.8). Married people or individuals in a stable relationship search the Internet more often for health information than do singles (OR: 1.9, 95% CI: 1.2–2.9). Also, heavy use of health-care services compared to non-use is associated with a higher likelihood of using the Internet for health-related matters (OR: 1.7, 95% CI: 1.2–2.5).

**Conclusions:**

In order to achieve equity in health, health-related Internet use by the socially deprived should be promoted through measures to increase their level of e-health literacy. Furthermore, longitudinal studies are needed in order to gain reliable data/results on determinants of health-related Internet use.

**Electronic supplementary material:**

The online version of this article (doi:10.1186/s12889-015-1423-0) contains supplementary material, which is available to authorized users.

## Background

Before the Internet, it was difficult for the lay public to access health information, which was found mainly in medical textbooks and journals [1]. Since the launch of the World Wide Web (WWW), the number of online health information seekers has grown remarkably. Today, the Internet represents a major source of health-related information. International studies show that in the USA up to 72% [2] and in Europe up to 71% [3] of Internet users conduct health-related searches. According to most studies, the main reasons for seeking health information on the Internet are specific diseases or health problems [2,4]. The literature suggests that, due to the increasing dissemination and use of online health information, patients have been empowered and the physician–patient relationship has become increasingly participatory [5,6]. This development has had positive consequences: Empirical studies have shown that informed patients are more compliant, which makes for better health outcomes [5,7,8]. Moreover, health costs can be reduced, as informed patients and citizens use health-care services in a more efficient manner [5,8].

Although concerns have been voiced regarding the quality of online health information, only a few cases of actual harm in connection with health information gleaned from the Internet have been reported [9-11] and the overall impact has been positive [3,8,12]. Negative health-related consequences are only likely if there is a combination of low confidence in the attending physician, a high readiness to self-medicate, inaccurate health information and deficits in evaluating the quality of health information [8].

Technically, searching for health information is referred to as “Health Information Seeking Behavior” (HISB). Despite the absence of a consistent definition, most authors describe HISB as an influencing factor or a component of health behavior [13].

Theorizing about the Internet as a communication medium for HISB is still in its early stages. Most authors point out the need for a multidisciplinary framework combining approaches from psychology, the health sciences, sociology, and the communication and media sciences to explain HISB. Most theories draw on existing theoretical frameworks, such as: the Theory of Planned Behavior, the Health Belief Model, Stress, Appraisal and Coping Theory, the Technology Acceptance Model and/or the Uses and Gratifications Approach [13-15].

Theories and empirical studies of the Internet as a medium used for HISB describe it as being determined by multiple factors. According to these, cognitive, media-related, health-related and sociodemographic factors interact in determining whether and how health-related information is sought on the Internet. The theories indicate an indirect/moderating effect of personal and contextual factors on HISB, while media-related and psychological factors are attributed a direct effect on HISB [13-15].

Cognitive factors mentioned in the literature include: perceived control of conduct, health consciousness, self-efficacy and perceived health risk. Perceived access, usability and reliability of the Internet are listed amongst the media-related factors. Health-related determinants include, *inter alia*, self-perceived health, (the severity of) health problems and the care dependency of relatives. Sociodemographic factors mentioned and investigated in the literature are: age, gender, education, income, employment status and others [3,11,12,16-34].

Most studies on influencing factors have been based on large-scale quantitative telephone surveys. They are almost exclusively cross-sectional. In most cases, regression analyses were performed to show the statistical probability of searching the Internet for health information. The majority of studies were conducted in the USA. So far, mostly sociodemographic and health-(care-)related determinants have been examined. Since the methods employed were not uniform, the study results are difficult to compare [12,16].

In the light of inequalities in health, the association between socio-economic status (SES) and HISB on the Internet is of particular importance and hence it is the focus of this study. In a survey of 4,500 adult Hong Kong Chinese, HISB on the Internet and in magazines was found to be a significant mediator between SES and self-rated health [35]. Evaluating the same survey data, a higher SES was also found to be a strong predictor of HISB on the Internet [36]. A European study with 12,000 participants reached similar conclusions: the researchers found SES to be linked to general Internet use, HISB on the Internet and subjective health. Moreover, they found Internet use to be a mediator between SES and subjective health [37]. A significant association between single SES dimensions (education, income, occupation) and HISB on the Internet has also been found in other studies from Australia, New Zealand, the USA and Saudi Arabia [11,18,38].

So far, only three studies have examined determinants of HISB on the Internet in Germany. One examined data from the “Media and Health Survey” by the Federal Ministry of Health in 2001 [39]. The other two used data collected in Germany for the “eHealth Trends in Europe” study in 2005 and 2007 [25,40].

In this study, hitherto unexamined socidemographic and health-(care-)related determinants of HISB on the Internet in Germany are examined (migration background, relationship status, health insurance status, use of health-care services). This study investigates the influence of migration background on health-related Internet use by looking at the country of birth and by differentiating between first- and second-generation migrants. Most previous international studies have only analyzed the impact of ethnicity on HISB on the Internet. Furthermore, the impact of social class on HISB on the Internet is investigated. In the majority of studies, this has only been done by looking at the single dimensions, but not on SES as a whole. The SES index used in this study takes into consideration the associations between the single dimensions of occupational status, income and education. Also, it is possible to reduce the proportion of missing values by substituting for them with the average values of the other two dimensions, which leads to more reliable results.

The purpose of this study is to generate a profile of users and non-users of online health information, in order to identify discriminating factors between users and non-users. In this way, hard-to-reach target groups can be detected.

## Methods

This study is based on a secondary data analysis of the NRW Health Survey LZG.NRW 2011, a cross-sectional survey of North Rhine–Westphalia (NRW). NRW is the most densely populated federal state of Germany with 17,571,856 inhabitants (December 2013), almost 22% of the overall German population.

### Data source

The NRW Health Survey is a telephone survey which is conducted once a year. It is representative of the adult population (>18 years) living in private households and using a fixed telephone line. It is conducted by the ‘Landeszentrum für Gesundheit’ (LZG.NRW). The LZG.NRW is the Centre for Health in North Rhine–Westphalia and serves the state government, *inter alia*, through health reporting. Based on the survey results, recommendations can be made regarding existing or planned target-group-specific prevention programs [41].

The 2011 survey included supplementary questions relating to the field of “Internet and Health” and these were assessed in the present study.

A two-stage sampling was conducted. First, households in NRW with a landline number were selected at random and the household member with the birthday latest in the year was interviewed. According to the Federal Bureau of Statistics in Germany, 90% of private households had a landline in 2013 and 93% of private households had at least one mobile phone. In 2011, 12% of the German population solely used mobile phones. In the present study only landline users were included, since mobile phone users cannot be limited to NRW. Eligibility criteria for participants were: living in NRW, possessing a landline (only private households, no business phones), being at least 18 years old and speaking German fluently. Interviews were conducted in May 2011 using the technique of computer-assisted telephone interviewing [42]. In order to ensure high survey quality, the questionnaire underwent a pretest, the interviewers were tutored and supervised, and ten attempts were made to contact individuals who were harder to reach (e.g. working people) [41].

### Research question and hypotheses

The main research question was:

What influences do health-(care-)related*and sociodemographic factors have on health information seeking on the Internet in NRW, Germany?

*use of health-care services (physician visits, hospital nights), self-perceived health, chronic disease(s)

Statistical hypotheses were generated based on a literature search for determinants of HISB on the Internet. The direction of differences in HISB on the Internet was assumed based on the results of previous studies for each determinant/independent variable (see discussion).

The hypotheses for bivariate analysis were that NRW residents differ in HISB on the Internet by…

- social class/socio-economic status (SES) (HISB on the Internet is more likely in higher social classes)
- gender (HISB on the Internet is more likely in women)
- age (HISB on the Internet is more likely in younger people)
- migration background (HISB on the Internet is more likely in people without migration background)
- relationship status (HISB on the Internet is more likely in people with a life partner)
- parental status (HISB on the Internet is more likely in people with children)
- employment status (HISB on the Internet is more likely in employed people)
- town size (HISB on the Internet is more likely in urban areas with a higher population density)
- self-perceived health (HISB on the Internet is more likely in people with poor self-perceived health)
- chronic diseases/conditions (HISB on the Internet is more likely in chronically ill people)
- health insurance status (HISB on the Internet is more likely in privately insured people)
- use of health-care services (HISB on the Internet is more likely in case of a heavy use of health care services)

The hypotheses for multivariate analysis were:

- There is an association between sociodemographic factors and HISB on the Internet in NRW.
- There is an association between health-(care-)related factors and HISB on the Internet in NRW.

### Literature search

To review the current state of research on determinants of HISB on the Internet, a literature search was conducted that included studies from all around the globe investigating influencing factors with regard to health-related Internet use in the general population or in the group of Internet users. Studies published in the English and German languages with a quantitative approach were included in the search. Another eligibility criterion was the definition of HISB on the Internet: Mainly studies assessing HISB on the Internet as a health-related use of the World Wide Web (search on websites) were included. Thus, as a rule, studies investigating online communication via email or chat for health purposes were not considered. This limitation was imposed in order to enhance their comparability with the present study.

### Study variables and statistical analysis

The dependent variable is binary and measured by the question “Do you use the Internet to search for information on medical or health issues? (yes/no)”. This question was only asked of those respondents who stated that they used the Internet (“Using the Internet at least now and then”). Thus, the subsample of Internet users (n = 1,488) was the basis for the bivariate and multivariate analyses. If the Internet users stated that they used the Internet in health matters, they were asked for the purposes/reasons. If they stated that they didn’t use the Internet in health matters, they were asked which other sources they drew on for health information.

The independent/explanatory variables have ordinal or nominal scaling and constitute two content-related groups: sociodemographic and health-(care-)related variables. The sociodemographic variables are: age, gender, social class, migration background, relationship status, parental status, employment status and town size. The health-(care-)related variables are: self-perceived health, chronic diseases/conditions, health insurance status and use of health-care services.

The independent variable ‘social class’ was generated based on Winkler’s social class index [43], comprising the three socio-economic dimensions ‘educational qualification’, ‘occupational status’ and ‘household net income’. Calculating the index, points on a scale from 1 to 7 were assigned for each dimension, 1 being the lowest and 7 the highest educational qualification/occupational status/household income group. The points from each dimension were then summed to yield a total score. Depending on the score, respondents were classified as belonging to the lower, middle or upper social class.

Some independent variables were reduced in category number (to a maximum of four) to generate more meaningful results. For example, in the variable ‘health insurance status’, the category “no insurance” was not included in the analyses as only 0.3% of respondents stated not having health insurance coverage. Regarding ‘parental status’, it should be pointed out that the variable only measures the number of children under the age of 15 who are living in a joint household with their parents.

For multivariate analysis, some variables were aggregated to generate more meaningful results and to avoid multicollinearity. The variables ‘country of birth’ and ‘parents’ country of birth’ were pooled into the variable ‘migration background’. The category “no migration background” comprises respondents born in Germany whose parents were also born in Germany. Respondents with one or both parents not born in Germany fall into the category “parental migration background”. The category “own migration background” includes respondents who were not born in Germany.

The variables ‘number of visits to the doctor in the last 3 months (except dentist)’ and ‘number of nights spent in a hospital as a patient in the last 12 months’ were pooled into the variable ‘use of health-care services’. No visits to the doctor and no nights spent at a hospital as a patient were defined as “no usage”, one visit and 1–7 nights in the hospital as “low usage”, and several visits and more than 8 nights in the hospital as “high usage”.

‘Marital status’ (married yes/no) and ‘partnership status’ (in a relationship yes/no) were pooled into the variable ‘relationship status’.

Regarding missing data, there were intentional/expected missing values in connection with those variables that were preceded by screening questions. The percentage of genuine missing values (“don’t know”, “no answer”) was below 5% for almost all variables. These cases were not included in the analyses. Only the household income variable showed a high percentage of missing values (27.7–35.5%). For the social class index, missing values were substituted by mean values when only two out of three values were available. (For further information on the assessment of all variables in the questionnaire that were investigated see Additional file 1).

In order to determine whether the variables were statistically independent or associated with the outcome variable, simple logistic regression analyses were performed. In this way, unadjusted odds ratios (ORs) were calculated. Confounding could be detected by comparing these unadjusted ORs with the adjusted ORs determined in the multivariate analyses.

Multiple logistic regression analysis was employed to examine the joint explanatory power of the independent variables on HISB on the Internet. Three models were tested: one including only sociodemographic variables, one including only health-(care-)related variables and one final model including all explanatory variables. The reference categories were picked so as to allow comparisons of extreme groups. The independent variables were entered in one block in all models. The final regression model shows the statistical probabilities of HISB on the Internet when sociodemographic and health-(care-)related factors are controlled for.

The preliminary test for multicollinearity showed variance inflation factor values below 10. Thus, multicollinearity could be excluded for the independent variables and all predictor variables were included in the multivariate analyses.

Those variables not showing a statistically significant association with the outcome variable in the bivariate analyses were also included in multivariate analysis to detect any apparent non-associations. These variables were also used as control variables.

The significance level for all statistical tests was set at *p* < .05. SPSS 18 (Statistical Package for Social Sciences) was used for the statistical analyses.

## Results

2,000 interviews were completed during the survey in 2011, amounting to a response rate of 74.4%. In order to avoid bias, the data set was weighted using the age and gender distributions from the ministerial statistics for the population of NRW in 2011.

### Descriptive results

Respondent ages ranged from 18 to 94 years, with an average age of 50 years. 48.1% of respondents are men, 51.9% are women. Table 1 (column “Total sample”) shows the distribution of sociodemographic and health-(care-)related variables for the total sample.

**Table 1:** **Sociodemographic and health-(care-)related sample characteristics (data in %)**

	**Online health information seekers (n = 1,010)**	**Internet users (n = 1,488)**	**Total sample (n = 2,000)**
**Gender**			
Male	50.9	53.1	48.1
Female	49.1	46.9	51.9
**Age (years)**			
18-29	20.0	22.2	16.7
30-44	29.5	28.6	22.9
45-59	33.3	31.5	28.5
60 and over	17.2	17.8	31.9
**Social class**			
Lower	13.0	17.8	24.8
Middle	46.3	46.8	47.0
Upper	40.8	35.3	28.2
**Migration background**			
Own migration background	10.5	12.7	13.7
Parental migration background	11.6	11.5	9.8
No migration background	77.9	75.8	76.5
**Relationship status**			
Unmarried and no partner	15.5	18.3	22.8
Married or partner	84.5	81.7	77.2
**Town size (inhabitants)**			
Under 20,000	5.5	6.1	6.0
Under 100,000	23.9	25.3	25.6
Under 500,000	32.1	33.9	33.7
500,000 or more	38.5	34.7	34.8
**Employment status**			
Full time	48.6	47.6	39.4
Part time	20.8	20.1	17.3
Unemployed	30.6	32.3	43.4
**Parental status**			
No children <15 years	66.5	65.6	70.5
1-2 children <15 years	30.3	29.5	25.3
3 or more children <15 years	3.2	4.9	4.2
**Self-perceived health**			
Very good/good	79.0	80.0	71.8
Moderate	16.2	15.7	20.5
Poor/very poor	4.9	4.3	7.7
**Chronic diseases/conditions**			
Yes	34.0	31.1	37.1
No	66.0	68.9	62.9
**Health insurance status**			
Statutory	80.7	81.7	83.1
Private	19.3	18.3	16.9
**Use of health care services**			
None	36.5	39.5	35.7
Low	32.0	32.7	32.8
High	31.5	27.9	31.5

74.4% of the interviewees are Internet users. Of these, 67.9% also use the Internet to search for health information.

51.6% of the interviewees reported seeking health-related information online because they are sick themselves (information on illness and treatment options). This is the main reason for health-related Internet searches. About one third of respondents (35.5%) search the Internet for health information not for themselves, but for another person who is sick. A fifth (21.4%) go online to look up symptoms, 6.1% to find alternative treatment methods, 5.6% for information on health-related topics that are discussed in the media. 2.7% of participants use the Internet to get a second medical opinion and to find information on health insurance options, respectively.

Of those respondents who do not use the Internet for health purposes, 47.2% use other information sources to gather health information (magazines, television, physician, pharmacy, etc.).

Table 1 shows the characteristics of online health information seekers by sociodemographic and health-(care-)related factors in comparison to the total sample and the sub-sample of Internet users.

### Bivariate results

Table 2 shows the results of the simple logistic regression analyses. Within the health-(care-)related variables, ‘chronic diseases/conditions’ are significantly associated with HISB on the Internet. In relation to the use of health-care services, only the comparison of the extreme groups “high usage” and “no usage” proved statistically significant. No significant association with HISB on the Internet was found for ‘self-perceived health’ or ‘health insurance status’.

**Table 2:** **Bivariate and multivariate associations of sociodemographic and health-(care-)related factors with HISB on the Internet**

**Explanatory variables OR [95% CI]**	**Bivariate logistic regressions**	**Multivariate model I**	**Multivariate model II**	**Final multivariate model**
		**n = 1,024**	**n = 1,420**	**n = 1,002**
		**R** ^**2**^ **= 0.129**	**R** ^**2**^ **= 0.032**	**R** ^**2**^ **= 0.151**
**Gender**				
Male (ref.)	1.00 (ref.)	1.00 (ref.)		1.00 (ref.)
Female	**1.31 [1.05-1.63]**	**1.63 [1.20-2.21]**		**1.52 [1.12-2.08]**
**Age (years)**				
18-29 (ref.)	1.00 (ref.)	1.00 (ref.)		1.00 (ref.)
30-44	**1.47 [1.09-2.00]**	1.08 [0.69-1.69]		0.98 [0.61-1.55]
45-59	**1.59 [1.18-2.15]**	0.95 [0.63-1.45]		0.88 [0.57-1.36]
60 and over	1.21 [0.86-1.69]	0.74 [0.45-1.22]		0.67 [0.40-1.12]
**Social class**				
Lower (ref.)	1.00 (ref.)	1.00 (ref.)		1.00 (ref.)
Middle	**2.12 [1.57-2.86]**	**1.98 [1.40-2.80]**		**2.24 [1.56-3.20]**
Upper	**3.83 [2.75-5.34]**	**3.51 [2.38-5.17]**		**4.04 [2.65-6.17]**
**Migration background**				
No migration background (ref.)	1.00 (ref.)	1.00 (ref.)		1.00 (ref.)
Own migration background	**0.55 [0.40-0.76]**	**0.53 [0.36-0.76]**		**0.56 [0.38-0.82]**
Parental migration background	0.96 [0.67-1.37]	1.09 [0.72-1.66]		1.17 [0.75-1.81]
**Relationship status**				
Unmarried and no partner (ref.)	1.00 (ref.)	1.00 (ref.)		1.00 (ref.)
Married or partner	**1.75 [1.33-2.29]**	**1.86 [1.20-2.87]**		**1.87 [1.19-2.93]**
**Parental status**				
No children <15 years (ref.)	1.00 (ref.)	1.00 (ref.)		1.00 (ref.)
1-2 children <15 years	1.04 [0.80-1.34]	1.00 [0.71-1.40]		1.08 [0.76-1.53]
3 or more children <15 years	**0.38 [0.23-0.64]**	0.56 [0.29-1.07]		0.67 [0.35-1.31]
**Employment status**				
Unemployed (ref.)	1.00 (ref.)	1.00 (ref.)		1.00 (ref.)
Full time	1.26 [0.98-1.61]	1.00 [0.69-1.45]		1.06 [0.72-1.55]
Part time	1.31 [0.96-1.79]	0.85 [0.56-1.31]		0.89 [0.57-1.38]
**Town size (inhabitants)**				
Under 20,000 (ref.)	1.00 (ref.)	1.00 (ref.)		1.00 (ref.)
Under 100,000	1.13 [0.70-1.81]	0.96 [0.55-1.67]		0.97 [0.55-1.72]
Under 500,000	1.14 [0.72-1.80]	1.07 [0.62-1.84]		1.01 [0.58-1.78]
500,000 or more	**1.92 [1.20-3.07]**	1.61 [0.93-2.81]		1.61 [0.91-2.85]
**Chronic diseases/conditions**				
No (ref.)	1.00 (ref.)		1.00 (ref.)	1.00 (ref.)
Yes	**1.53 [1.2-1.96]**		**1.37 [1.04-1.81]**	1.17 [0.84-1.65]
**Self-perceived health**				
Very good/good (ref.)	1.00 (ref.)		1.00 (ref.)	1.00 (ref.)
Moderate	1.16 [0.86-1.58]		0.92 [0.66-1.29]	1.09 [0.73-1.61]
Poor/very poor	1.61 [0.89-2.91]		1.31 [0.65-2.63]	1.38 [0.61-3.16]
**Health insurance status**				
Statutory (ref.)	1.00 (ref.)		1.00 (ref.)	1.00 (ref.)
Private	1.22 [0.91-1.64]		1.34 [0.99-1.81]	0.95 [0.64-1.42]
**Use of health care services**				
None (ref.)	1.00 (ref.)		1.00 (ref.)	1.00 (ref.)
Low	1.18 [0.92-1.52]		1.09 [0.84-1.41]	1.01 [0.74-1.38]
High	**1.99 [1.50-2.64]**		**1.78 [1.31-2.44]**	**1.73 [1.19-2.51]**

ref. = reference category, OR = odds ratio, CI = confidence interval, **numbers in bold =** ***p*** **< .05**.

With regard to the sociodemographic variables, the simple logistic regressions showed a statistically significant association with HISB on the Internet for ‘gender’, ‘relationship status’ and ‘social class’. For ‘age’, significant associations were found for all category comparison, except for one (“60 years and older” vs. “18-29 years”). As for ‘town size’, ‘migration background’ and ‘parental status’, significant associations with the outcome variable were found only for the comparisons of extreme groups. No significant association was found for ‘employment status’.

### Multivariate results

Table 2 shows the results of the multiple logistic regressions. The regression model including the sociodemographic variables accounts for 12.9% of the variance in the outcome variable, while the model including the health-(care-)related variables explains 3.2%. The final model has the strongest explanatory power, explaining 15.1% of variance (measured using Nagelkerke’s R^2^).

#### Sociodemographic variables

A significant association was found for ‘social class’ and HISB on the Internet in both the first and the final multivariate regression model. People from the middle class are 2.24 times more likely to search the Internet for health-related information than are people from the lower class (OR: 2.24, 95% CI: 1.56–3.20). The largest difference was found in the comparison of extreme groups, with members of the upper class being 4.04 times more likely than members of the lower class to seek health information on the Internet (OR: 4.04, 95% CI: 2.65–6.17).

The first and the final multivariate regression models consistently show a significantly higher probabilitiy of HISB on the Internet for women compared to men. The final regression model reveals that, adjusted for sociodemographic and health-(care-)related variables, women are 52% more likely to search the Internet for health information than men (OR: 1.52, 95% CI: 1.12–2.08).

Regarding the impact of migration experience on HISB on the Internet, the multivariate regression models show statistically significant differences between the extreme groups “own migration background” and “no migration background”. The final regression indicates that people with their own migration experience are 44% less likely to use the Internet to seek health information than people without migration experience (OR: 0.56, 95% CI: 0.38–0.82).

The multivariate analyses also show a significantly greater statistical probability of using the Internet for health purposes amongst respondents with a spouse/partner. According to the final regression model, married people or people in a stable relationship are 87% more likely to search the Internet for health information than are singles (OR: 1.87, 95% CI: 1.19–2.93).

As the results of the multivariate analyses (Table 2) imply, no significant association with HISB on the Internet was found for the sociodemographic variables ‘parental status’, ‘age’, ‘employment status’ or ‘town size’.

Regarding ‘parental status’, people living with three or more children under the age of 15 tend to demonstrate a decreased statistical probability of HISB on the Internet compared with people living without children under the age of 15.

In terms of ‘age’, the multivariate results indicate that people over 30 are slightly less likely than those aged 18–29 to seek health information on the Internet.

Regarding ‘employment status’, individuals with part-time work seem to search the Internet for health information a little bit less often than the unemployed.

As for ‘town size’, the multivariate results show a higher statistical probability of HISB on the Internet for people living in larger cities than for people living in small towns.

#### Health-(care-)related variables

Regarding the ‘use of health-care services’, the multivariate analyses expose a significant association with the outcome variable for the comparison of the extreme groups “high usage” and “no usage”. According to the final regression model, heavy users of health-care services are 1.73 times more likely to seek health information on the Internet than non-users (OR: 1.73, 95% CI: 1.19–2.51).

For ‘chronic diseases/conditions’, the second regression model yields a significantly higher probability of HISB on the Internet amongst chronically ill compared to healthy respondents (OR: 1.37, 95% CI: 1.04–1.81), although the difference does not reach statistical significance in the final model.

Regarding ‘self-perceived health’, neither of the two multivariate models shows a statistically significant association with HISB on the Internet. However, in comparison to a “very good/good health status”, respondents who rate their health as “poor/very poor” seem to be more likely to search the Internet for health information.

The explanatory variable ‘health insurance status’ is not significantly associated with HISB on the Internet either. The results do not show a clear tendency as to whether statutorily or privately insured people are more likely to seek health information on the Internet.

Of the tested independent variables, ‘social class’ shows the greatest effect on HISB on the Internet (measured using ORs). In order to examine the influence of social class more precisely, a multiple logistic regression analysis was done with the three variables that constitute Winkler’s social class index: ‘educational qualification’, ‘occupational status’ and ‘household net income’. The results of this regression analysis (Table 3) show a non-significant influence for ‘household net income’, but a significant one for ‘education’ and ‘occupational status’. In terms of effect size, ‘educational qualification’ and ‘occupational status’ show comparable ORs: With every point gained on the respective scale, the statistical probability of HISB on the Internet rises 11% for ‘educational qualification’ and 17% for ‘occupational status’.

**Table 3:** **Multivariate associations of social class dimensions with HISB on the Internet (n = 956, R** ^**2**^ **= 0.062)**

**Explanatory variables**	**OR [95% CI]**
Education	**1.11 [1.03-1.21]**
Occupational status	**1.17 [1.07-1.29]**
Household net income	1.08 [0.99-1.19]

OR = odds ratio, CI = confidence interval, **numbers in bold =** ***p*** **< .05**.

## Discussion

Looking at the percentage of variance explained by the multivariate regression models, sociodemographic factors seem to exert a considerably greater influence on HISB on the Internet than do health-(care-)related factors. Yet, this greater explanatory power may in part be due to the higher number of variables included (8 sociodemographic vs. 4 health-(care-)related). The final regression model, comprising all independent variables, explains 15.1% of the variance in seeking health information online. Since this explanatory power is rather small, it would appear that other determinants play a more decisive role in HISB on the Internet. The proportion of explained variance in HISB on the Internet, depending on various explanatory variables, as determined in several international studies, amounts to 17–43% [26].

One reason for the small explanatory power of this study could be that health-(care-)related determinants such as acute illness, disease of a loved one, satisfaction with information from physicians and medication intake were not considered [17,44,45]. Moreover, and more importantly, media-related and cognitive/psychological determinants of HISB on the Internet were not considered at all in this study.

The final regression model demonstrates a significant influence from ‘gender’, ‘relationship status’ and ‘social class’ and a partly significant influence from ‘migration background’ and ‘use of health-care services’ on HISB on the Internet. ‘Social class’ proved to be the strongest predictor of HISB on the Internet.

The statistical analyses in this study reveal that people from the upper and middle social classes are more likely to be online health information seekers than those with a lower social class background. This circumstance may be explained by differences in e-skills and health literacy. According to the WHO, health literacy encompasses the cognitive and social skills required to access, understand and use health information effectively to promote good health [46]. E-skills include media-related skills (navigating through the WWW) and information-related skills (search strategies) [47]. Several studies have reported poorer e-skills in people from lower social classes [48,49]. Other studies reported a significantly higher probability of HISB on the Internet amongst people with good compared to bad e-skills [44,47]. Some studies have reported a lower level of health literacy for the lower social classes [49,50]. The European Health Literacy Survey (HLS-EU) collected data from eight European countries, including a sample from NRW, Germany, in 2011. The findings display a positive association between high education/social status and health literacy [51].

A study from the USA showed that a high level of health literacy correlated significantly with greater use of an online-based information portal by diabetes patients [52], and an Australian study reported a fourfold increase in HISB on the Internet amongst older people with a high level of health literacy [53]. This context could also explain the lower frequency of seeking health information on the Internet by the socially deprived that was found in the present study. Most previous studies only considered individual dimensions of social class (income, education, occupational status). The state of research points to a higher probability of HISB on the Internet amongst people with higher household incomes and educational qualifications [29,36-38,45,54], which corresponds with the results of the present study.

Regarding ‘gender’, the statistical analyses recognize women as being more likely to search for health topics online than men. A possible explanation is that women usually take responsibility for health-related issues in partnerships and families due to socialization and learned gender roles [8,26,55]. Hence, women probably look up health topics online not only for themselves, but also for a partner, parent and/or child and are therefore more likely than men to show HISB on the Internet. This might especially apply to the nursing of relatives, as the majority of informal caregivers are women [56]. Furthermore, women are generally more health-conscious than men, which could explain their higher use of online health information [8,55,57]. Empirical evidence from two studies supports these assumptions [44,58]: There is a significantly higher statistical probability of HISB on the Internet if health awareness is high. Previous international studies have predominantly indicated a significantly higher probability of seeking health information online amongst women as opposed to men [19,30,44,45]. These findings agree with the present study.

The association between HISB on the Internet and migration background detected in the present study could be ascribed to cultural factors. According to the Federal Bureau of Statistics in Germany, Turks form the largest group of migrants, making up 18.5% of all migrant groups. According to the federal state agency IT.NRW Turks also form the largest group of migrants and foreigners in NRW. Several German studies have found that Turkish migrants possess rather traditional health-related beliefs and tend to attribute illnesses to external, fatalistic causes (destiny, age, genes). These health-related ideas go along with a reduced self-responsibility and a rather passive/less preventive attitude toward health behaviors. Yet, the overall group of migrants in NRW and Germany is very heterogenous. It is therefore unclear and remains to be investigated further, whether the findings on Turks also apply to other migrant groups [59-61]. Language difficulties should not be an issue, as the Internet provides information in practically every language. Most studies on the influence of ethnicity on HISB on the Internet have been conducted in the USA and indicate that white Americans are more likely than Afro-Americans or Hispanics to search the Internet for health information [12,16,19]. Due to the different methods used, the results are not readily comparable with the present study.

The present study also revealed that married couples or people in a stable relationship are more likely to conduct online health searches than singles. This is probably due to stronger social ties in relationships, as people in a relationship seek health information on the Internet not only for themselves, but also for their spouses or partners. This assumption is supported by previous findings from the Pew Internet & American Life Project, indicating that more surrogate seekers were married or had children compared to those who sought health information only for themselves [28]. However, research on the influence of relationships/marriage on HISB on the Internet is, on the whole, inconsistent. In contrast to the present and other studies [30,44,45,62], Siliquini et al. [63] found a higher probability of HISB on the Internet for people living alone (singles, widowed and divorced people) compared to people in a steady relationship or married people.

In the present study, HISB on the Internet is positively influenced by a heavy use of health-care services. One explanation could lie in physician–patient communication. Possible communication problems, such as incomprehensible medical jargon, may provoke a demand for further and more comprehensible health information, as was also reported by the Health Information National Trends Survey in the USA [54]. It was found that e-health users felt significantly less well informed and included by their physician than non-users. Similarly, a French study reported that difficulties in understanding a physician significantly increased the probability of HISB on the Internet [45]. Another interpretation of this finding could be that people who are ill actually engage more in illness compared to those who are well, meaning they both see a doctor and go on the Internet - as two strategies of health information seeking. The findings of some earlier studies examining the association between HISB on the Internet and the use of health-care services agree with the results of this study [3,30,63]. Yet, Koch-Weser et al. [54] and AlGhamdi & Moussa [18] found that a lower or moderate number of physician visits was associated with an increased statistical probability of HISB on the Internet.

No significant association with HISB on the Internet was found for ‘age’, ‘employment status’, ‘parental status’, ‘town size’, ‘chronic diseases/conditions’, ‘self-perceived health’ or ‘health insurance status’ in the final multivariate regression model of the present study.

The variable ‘parental status’ does not measure parenthood appropriately, as only people with children up to the age of 14 who are living at home are considered parents. This may have skewed the results and might therefore explain why no significant association was found. Some previous studies have reported results that disagree with this study’s results on the influence of parenthood on HISB on the Internet [45,54]. In agreement with the results of this study, Atkinson et al. [19] found that a large number of children was associated with a decreased probability of HISB on the Internet.

In terms of employment status, the current state of research is also inconsistent. Some studies found employed people to be more likely to seek health information on the Internet [18,24,30]. In contrast to this, Andreassen et al. [3] reported that unemployed people have a greater probability of HISB on the Internet. In the present study, the unemployed are sociodemographically heterogenous, which could explain why no significant difference regarding HISB on the Internet was found between the employed and the unemployed.

Regarding chronic diseases/conditions, the majority of previous studies showed that chronically ill people were more likely to search for health information on the Internet than were healthy people [3,28,63]. The fact that no significant association was found in the present study could be explained not only by the fact that there are both “illness seekers” and “wellness seekers” [64], but also because not just the mere presence of a disease, but also the self-perceived health risk, is crucial in deciding for or against HISB on the Internet.

Empirical findings on the influence of health insurance status on HISB on the Internet are contradictory: Flynn et al. [44] found an increased probability of HISB on the Internet for statutorily insured people. In contrast, Ayers & Kronenfeld [17] reported a higher probability amongst people with private health insurance. Some authors argue that differences in HISB on the Internet by health insurance status are due to out-of-pocket payments [21]. This might be true for the USA, where out-of-pocket payments are costly and pose an obstacle to the use of health-care services for people without health insurance or with statutory (as opposed to private) health insurance. Yet, this is not the case in Germany, which has nationwide health insurance coverage and where out-of-pocket payments are not as high as in the USA [65,66]. This would explain why no significant association was found in the present study.

Findings from previous empirical studies on the association of age and self-perceived health with HISB on the Internet were inconsistent. Regarding self-perceived health, Rice [62] and Xiao et al. [31] found a higher probability for HISB on the Internet for bad subjective health. Ybarra & Suman [32], Sadasivam et al. [28] and Dumitru et al. [25] reached opposite conclusions. With respect to age, Ybarra & Suman [32] reported middle-aged people to be more likely than younger people to search for health information on the Internet. Koch-Weser et al. [54] on the other hand found that the probability for HISB on the Internet decreased with increasing age.

In terms of the influence of town size/population density on HISB on the Internet, most studies show that people in urban areas are more likely to search the Internet for health information than people in rural areas [12,20,44]. It remains to be elucidated why no significant associations between HISB on the Internet and age, self-perceived health or town size were found in the present study.

### Limitations

The present study has some limitations. The essential methodological limitation is the cross-sectional design, which does not allow causal conclusions. Other limitations lie in the eligibility criteria for participants: Only residents with a landline were interviewed, which excludes residents who only use mobile phones. This could have biased the structure of study participants. Moreover, speaking fluent German was a requirement that could have influenced (reduced) the proportion of participants with a migration background. In addition, the question addressing health information seeking on the Internet was not formulated precisely, leaving it uncertain as to whether a computer and/or mobile phone was used as a device to seek health information on the Internet.

## Conclusions

The most important result of the present study is that belonging to a higher social class is a positive predictor of HISB on the Internet in NRW. As international studies have come to similar findings regarding single dimensions of social class, this seems to represent a global public health issue.

Several studies have shown a status gradient in health-relevant behavior, in the sense that health-damaging behavior is more frequent amongst the lower social classes [67]. Despite being in greater need of health information, lower social classes seek it the least. Thus, in order to reduce inequalities in health rather than reinforcing them, the disparities in HISB on the Internet must be addressed by adequate public health efforts [68,69]. A German study reported that socially deprived people search less for health information regardless of the communication medium [57]. This underlines the need for action to promote of HISB amongst the lower social classes, no matter what medium is used. A relevant measure in terms of HISB on the Internet would be promoting user skills, particularly e-health literacy amongst the socially deprived. The concept of e-health literacy encompasses the core areas of literacy, health literacy, information literacy, scientific literacy, media literacy and computer literacy [70]. Measures for socially deprived people with migration experience should be adapted for cultural particularities.

In addition to these practical recommendations, further research is needed. Longitudinal studies with primary data acquisition are necessary. For Germany, nationwide studies are required in order to gather representative results. In addition, dual sampling of landlines and mobile phones is needed in future telephone surveys.

It is well-documented that there is an association between migration background and lower SES [61]. In the present study, SES was controlled for in the analysis. Thus, differences in HISB on the Internet due to migration background cannot be ascribed to the effect of SES on HISB on the Internet. However, given the fact that the link between migration, ethnicity and SES is rather complex, this should be investigated in more detail in relation to HISB (on the Internet) in further research.

Furthermore, the results of this study raise questions as to how the differences in HISB on the Internet can be explained. Therefore, the explanations given in this paper need to be investigated further.

## Additional file

Additional file 1:
**Assessment of variables.** The table illustrates the assessment of the investigated variables in the questionnaire (including the original questions and categories of evaluation).

## Abbreviations

SES: Socio-economic status
HISB: Health Information Seeking Behavior
LZG.NRW: Landeszentrum Gesundheit Nordrhein-Westfalen (NRW Centre for Health)
NRW: North Rhine-Westphalia
