# Supplementary material for: Sociodemographic and health-(care-)related characteristics of online health information seekers: a cross-sectional German study
Source: BMC Public Health. 2015 Jan 29;15:31. doi: 10.1186/s12889-015-1423-0 (PMC4312446; doi:10.1186/s12889-015-1423-0)
Supplement: Additional file 1: — Assessment of variables. The table illustrates the assessment of the investigated variables in the questionnaire (including the original questions and categories of evaluation). [file 12889_2015_1423_MOESM1_ESM.pdf]

Additional file 1: Assessment of variables

Variable	Assessment in Questionnaire	Categories of Evaluation
General Internet use	Do you use the Internet, at least now and again (for any reason whatsoever)?	Yes<br>No
HISB on the Internet	Do you use the Internet to search for information on medical or health issues?	Yes<br>No
Reasons for HISB on the Internet	For which purposes do you search for health information on the Internet?	• I am sick (information on illness and treatment options)<br>• Another person is sick (information on illness and treatment options)<br>• Look up symptoms<br>• Alternative treatments<br>• Health-related topic discussed in the media<br>• Second medical opinion<br>• Information about health insurance<br>• Others
Use of other sources for health information (Internet excluded)	Do you use other sources to search for information on medical or health issues?	Yes<br>No
Sources for health information (Internet excluded)	Which other sources do you use?	• Physician<br>• Pharmacy<br>• Health insurance company<br>• Family/friends<br>• Magazines<br>• Professional literature<br>• Television<br>• Others
Age (calculated)	In which year and month are you born?	18-29 years<br>30-44 years<br>45-59 years<br>60 years and over
Gender	Please state your gender	male<br>female
Parents' country of birth	Is one of your parents, mother or father, not born in Germany?	Yes, my …<br>No
Country of birth	Are you born in Germany?	Yes<br>No
Employment status	Are you currently employed? If so, to which extent of working hours?	• No<br>• Full-time (35 hours/week and more)<br>• Part-time (under 35 hours/week)
Parenthood	How many people under the age of 15 are living in your household permanently?	0<br>1-2<br>3 or more
Marital status	What is your marital status?	Married<br>Divorced<br>Widowed<br>Single
Partnership status	Do you have a life partner?	Yes<br>No
Town size (calculated)	Where do you live (town)?	
Self-perceived health	How would you describe your health status in general?	Very good<br>Good<br>Moderate<br>Poor<br>Very poor
Chronic disease(s)	Do you suffer from one or several chronic diseases? (Annotation: They are long-term illnesses that require permanent medical care and control)	Yes<br>No
Health insurance status	Do you have a statutory or private health insurance?	Private<br>Statutory<br>No insurance

Physician visits	Did you visit a resident physician as a patient (except for dentists) in the last 3 months?	No<br>Yes, once<br>Yes, several times
Hospital nights	How many nights did you spend in a hospital as a patient in the last 12 months?	0 nights<br>1-7 nights<br>8 nights or more
Aggregated variables	• *Migration background:* country of birth & parents' country of birth<br>• *Use of health care services:* physican visits & hospital nights<br>• *Relationship status:* marital status & partnership status<br>• *Social class:* educational attainment (graduation, training) & household income & occupational position	
